# Synergistic effects of eukaryotic co-expression plasmid-based STAT3-specific siRNA and LKB1 on ovarian cancer *in vitro* and *in vivo*

**DOI:** 10.3892/or.2021.8149

**Published:** 2021-07-21

**Authors:** Yuan Pan, Liqun Zhang, Xinyue Zhang, Ruizhi Liu

Oncol Rep 33: 774-782, 2015; DOI: 10.3892/or.2014.3623

Following the publication of this paper, it was drawn to the Editors attention by a concerned reader that certain of the western blotting data shown in Figs. 1C and 6D bore unexpected similarities to data appearing in different form in other articles by different authors. Owing to the fact that some of the contentious data in the above article had already been published elsewhere, or were already under consideration for publication, prior to its submission to *Oncology Reports*, the Editor has decided that this paper should be retracted from the Journal. The authors agree with the decision to retract the paper. The Editor apologizes to the readership for any inconvenience caused.

